# Supplementary material for: Miro2 tethers the ER to mitochondria to promote mitochondrial fusion in tobacco leaf epidermal cells
Source: Commun Biol. 2020 Apr 3;3:161. doi: 10.1038/s42003-020-0872-x (PMC7125145; doi:10.1038/s42003-020-0872-x)
Supplement: Supplementary file 1 — Supplementary Information [file 42003_2020_872_MOESM1_ESM.pdf]

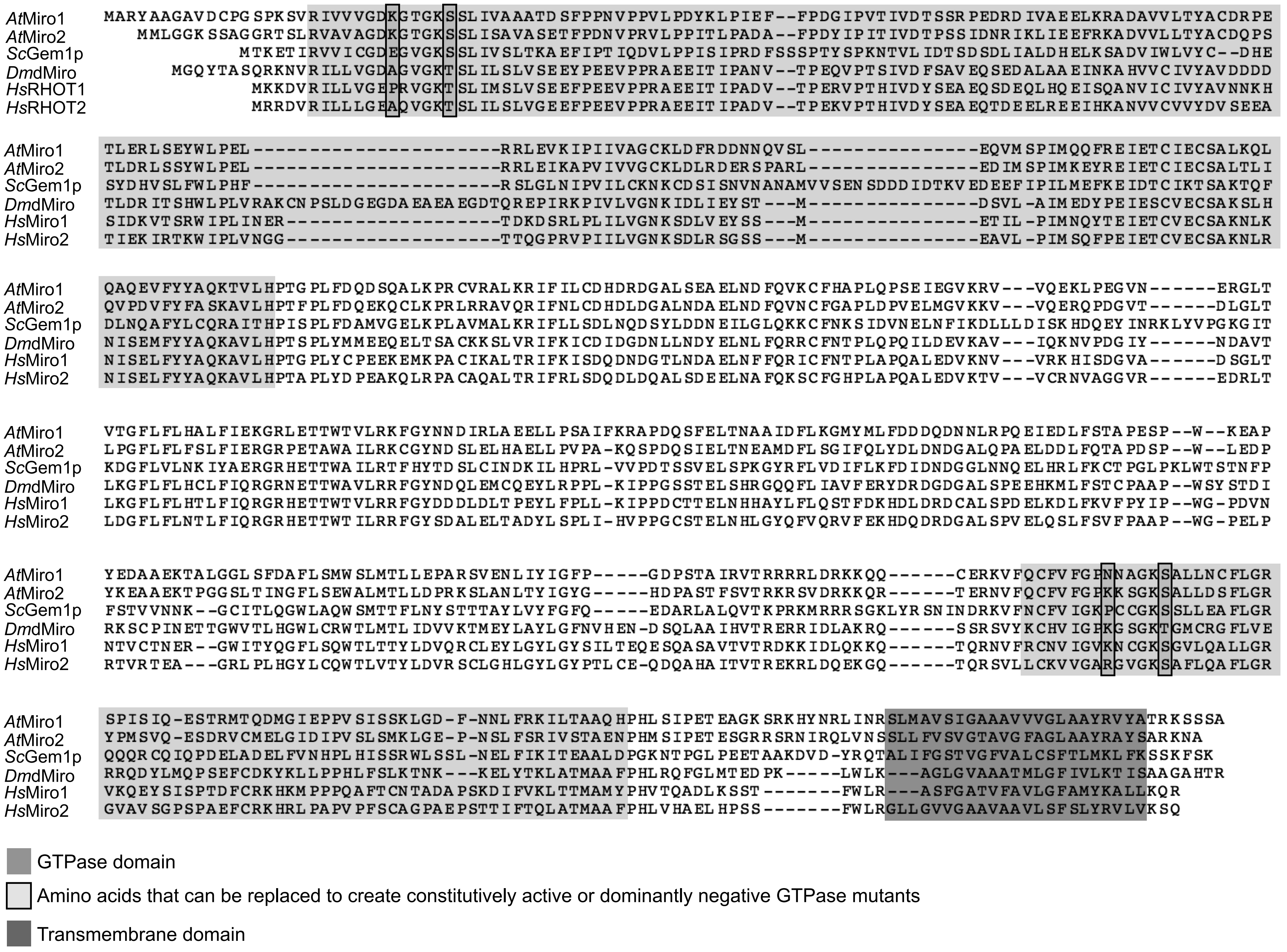

Supp Fig 1.

Alignment of Miro homologues: *Arabidopsis* (*At*Miro1, *At*Miro2) *S. cerevisiae* (*Sc*Gem1p), *Drosophila* (*Dm*dMiro) and human (*Hs*Miro1, *Hs*Miro2).

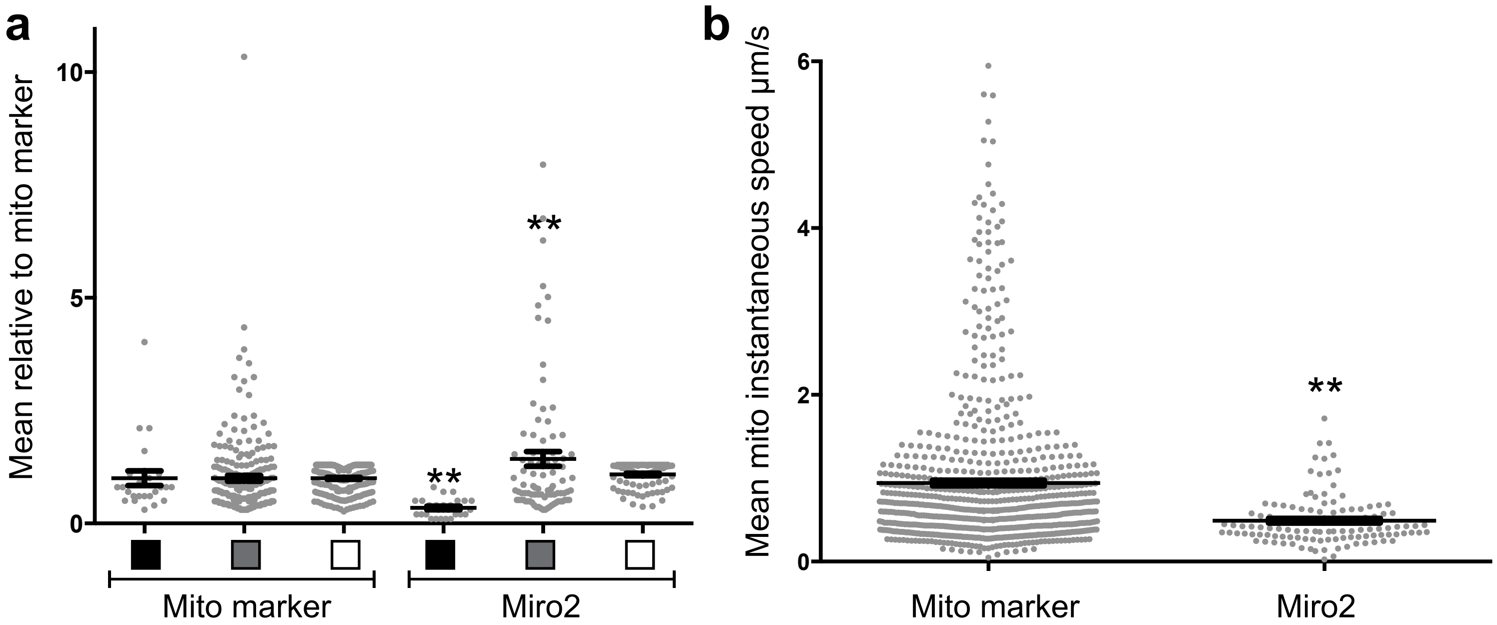

Supp Fig 2.

Effects of Miro2 on mitochondrial morphology, number, circularity and speed.

Bar charts of the mean with SEM (Fig 2) overlaid on to the entire dataset are indicated in Supp Figure 2. Data is normalised to mitochondrial marker control for ease of interpretation across several parameters. (a) Colour scheme is identical to figure 2 with mitochondrial number (black box), area (µm2, grey box), and circularity (white box) depicted. (b) Mean instantaneous speed (µm/s). Data taken from 3 independent experiments and analysed using a t-test with welches correction, **p< 0.02.

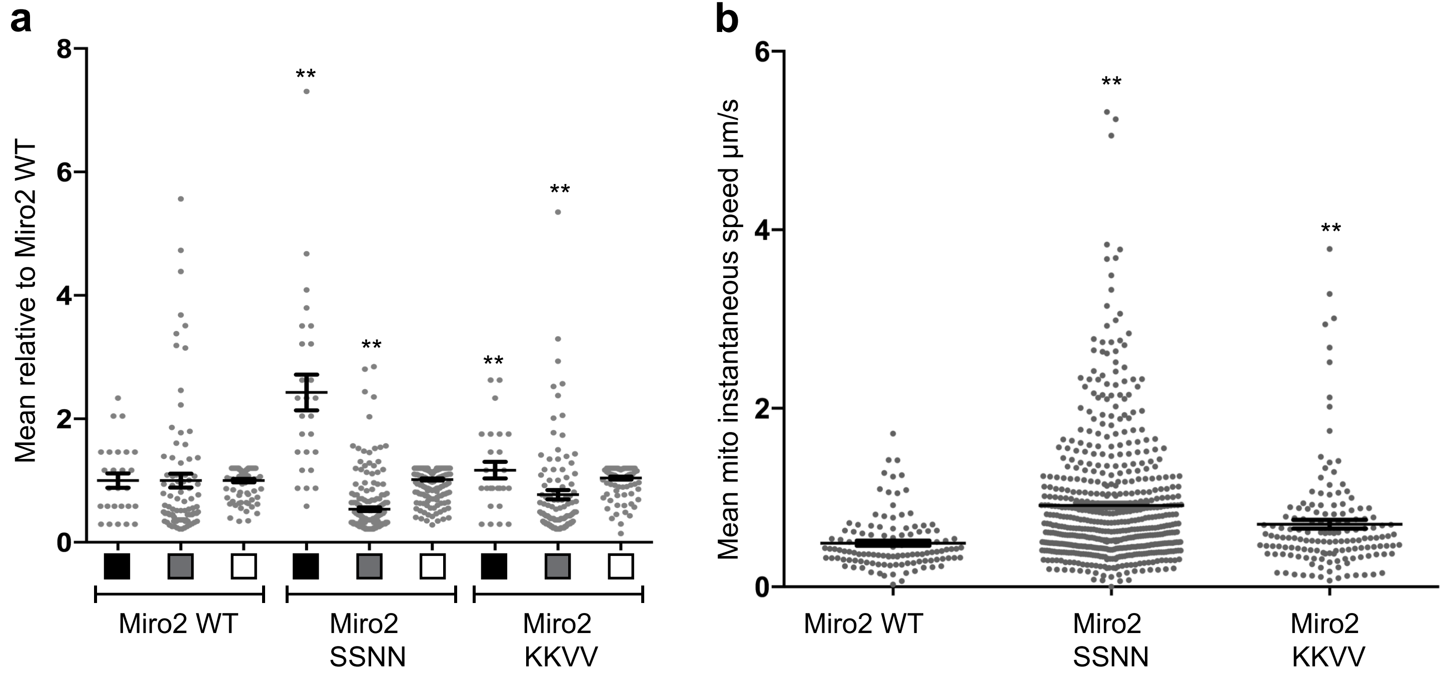

Supp Fig 3.

Effects of Miro2 GTPase mutants on mitochondrial morphology, number, circularity and speed.

Bar charts of the mean with SEM (Fig 3) overlaid on to the entire dataset are indicated in Supp Figure 3. Data is normalised to Miro2 WT for ease of interpretation across several parameters. (a) Colour scheme is identical to figure 3 with mitochondrial number (black box), area (µm2, grey box), and circularity (white box) depicted. (b) Mean instantaneous speed (µm/s). Data taken from 3 independent experiments and analysed using a t-test with welches correction, **p< 0.02.

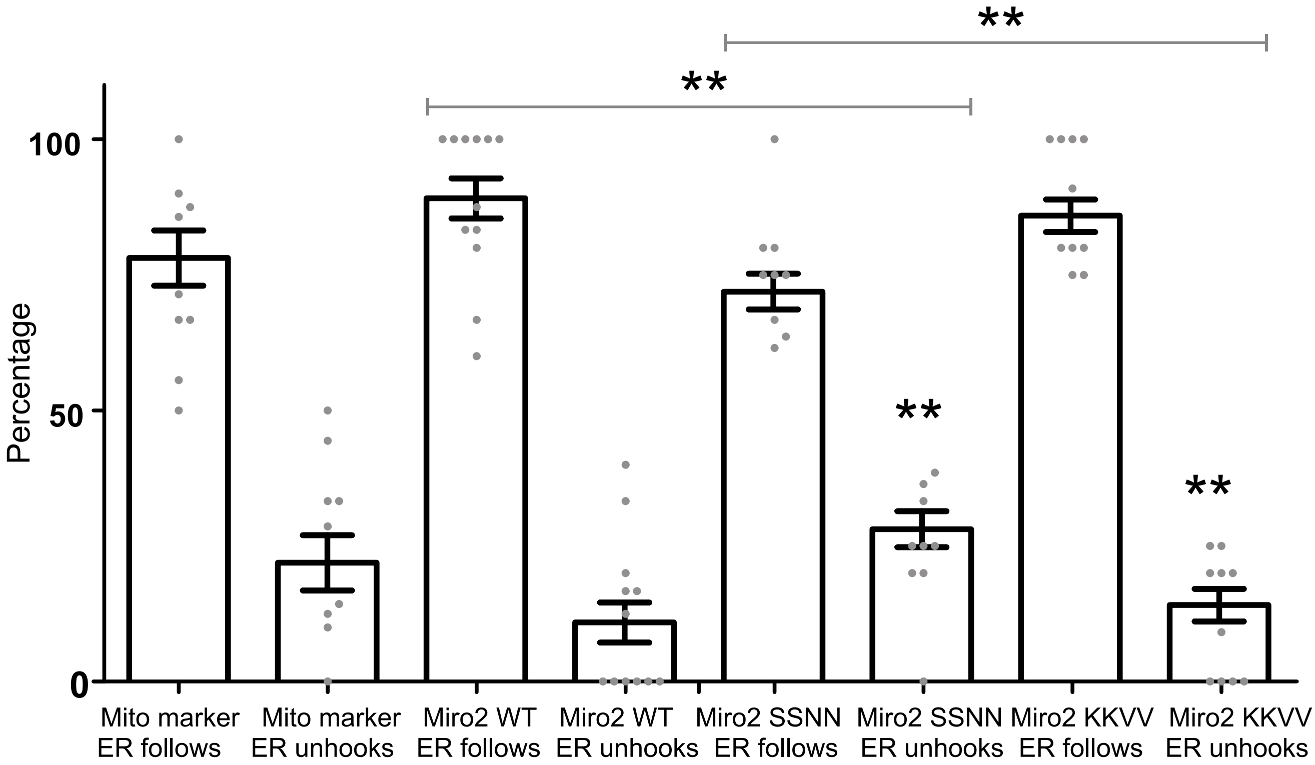

Supp Fig 4.

Mitochondrial tethering to the ER is affected by AtMiro2

Bar charts of the mean with SEM (Fig 4) overlaid on to the entire datasets are indicated in sup fig 4.

Data were analysed using a welches t-test of weighted means, **$p<0.01$.
