# Supplementary material for: Miro2 tethers the ER to mitochondria to promote mitochondrial fusion in tobacco leaf epidermal cells
Source: Commun Biol. 2020 Apr 3;3:161. doi: 10.1038/s42003-020-0872-x (PMC7125145; doi:10.1038/s42003-020-0872-x)
Supplement: Supplementary file 9 — Description of Additional Supplementary Material [file 42003_2020_872_MOESM9_ESM.pdf]

Description of additional supplementary items

Supplementary Movie 1. Movement of mitochondria over the ER in tobacco leaf epidermal cells

Spinning disc confocal microscopy movie of live tobacco leaf epidermal cells transiently expressing the mitochondrial inner membrane marker (magenta), and ER luminal marker (green). Scale bar indicates 5 µm, time is indicated in seconds (s).

Supplementary Movie 2. Optical trapping showing mitochondria are physically attached to the ER

TIRF microscopy movie of live tobacco leaf epidermal cells transiently expressing the mitochondrial inner membrane marker (green), and ER luminal marker (magenta). A mitochondrion is optically trapped and moved 6 µm at 6 µm/s. Figure 1 depicts three individual frames from this movie. Scale bar indicates 5 µm, time is indicated in seconds (s). Cells were treated with latrunculin b.

Supplementary Movie 3. Optical trapping showing mitochondria can be physically detached from the underlying ER.

TIRF microscopy movie of live tobacco leaf epidermal cells transiently expressing the mitochondrial inner membrane marker (green), and ER luminal marker (magenta). A mitochondrion is optically trapped and moved 6 µm at 6 µm/s. Figure 1 depicts three individual frames from this movie. Scale bar indicates 5 µm, time is indicated in seconds (s). Cells were treated with latrunculin b.

Supplementary Movie 4. Mitochondria movement in tobacco leaf epidermal cells expressing mitochondria marker

Spinning disc confocal microscopy movie of live tobacco leaf epidermal cells transiently expressing the mitochondrial inner membrane marker. Scale bar indicates 2 µm, time is indicated in seconds (s).

Supplementary Movie 5. Mitochondria movement in tobacco leaf epidermal cells coexpressing mitochondria marker and GFP-AtMiro2 WT

Spinning disc confocal microscopy movie of live tobacco leaf epidermal cells transiently coexpressing the mitochondrial inner membrane marker and GFP-AtMiro2 WT. Scale bar indicates 2 µm, time is indicated in seconds (s).

Supplementary Movie 6. Mitochondria movement in tobacco leaf epidermal cells coexpressing mitochondria marker and GFP-AtMiro2 SSNN

Spinning disc confocal microscopy movie of live tobacco leaf epidermal cells transiently coexpressing the mitochondrial inner membrane marker and GFP-AtMiro2 SSNN. Scale bar indicates 2 µm, time is indicated in seconds (s).

Supplementary Movie 7. Mitochondria movement in tobacco leaf epidermal cells coexpressing mitochondria marker and GFP-AtMiro2 KKVV

Spinning disc confocal microscopy movie of live tobacco leaf epidermal cells transiently coexpressing the mitochondrial inner membrane marker and GFP-AtMiro2 KKVV. Scale bar indicates 2 µm, time is indicated in seconds (s).

Supplementary data

Raw data used to calculate mitochondria number, area, circularity, speed and trapping characteristics shown in figures 2, 3 and 4 and supplementary figures 2, 3 and 4. Note, the figures show normalised data points.
